# PIAS1 is a crucial factor for prostate cancer cell survival and a valid target in docetaxel resistant cells

**DOI:** 10.18632/oncotarget.2658

**Published:** 2014-12-24

**Authors:** Martin Puhr, Julia Hoefer, Hannes Neuwirt, Iris E. Eder, Johann Kern, Georg Schäfer, Stephan Geley, Isabel Heidegger, Helmut Klocker, Zoran Culig

**Affiliations:** ^1^ Experimental Urology, Department of Urology, Medical University of Innsbruck, Innsbruck, Austria; ^2^ Department of Internal Medicine IV (Nephrology and Hypertension), Medical University of Innsbruck, Innsbruck, Austria; ^3^ Oncotyrol Laboratory for Tumor Biology and Angiogenesis, Innsbruck, Austria; ^4^ Division of Molecular Pathophysiology, Innsbruck Biocenter, Medical University of Innsbruck, Innsbruck, Austria

**Keywords:** prostate cancer, PIAS1, docetaxel, chemotherapy resistance, apoptosis

## Abstract

Occurrence of an inherent or acquired resistance to the chemotherapeutic drug docetaxel is a major burden for patients suffering from different kinds of malignancies, including castration resistant prostate cancer (PCa). In the present study we address the question whether PIAS1 targeting can be used to establish a basis for improved PCa treatment. The expression status and functional relevance of PIAS1 was evaluated in primary tumors, in metastatic lesions, in tissue of patients after docetaxel chemotherapy, and in docetaxel resistant cells. Patient data were complemented by functional studies on PIAS1 knockdown *in vitro* as well as in chicken chorioallantoic membrane and mouse xenograft *in vivo* models. PIAS1 was found to be overexpressed in local and metastatic PCa and its expression was further elevated in tumors after docetaxel treatment as well as in docetaxel resistant cells. Furthermore, PIAS1 knockdown experiments revealed an increased expression of tumor suppressor p21 and declined expression of anti-apoptotic protein Mcl1, which caused diminished cell proliferation and tumor growth *in vitro* and *in vivo*. In summary, the presented data indicate that PIAS1 is crucial for parental and docetaxel resistant PCa cell survival and is therefore a promising new target for treatment of primary, metastatic, and chemotherapy resistant PCa.

## INTRODUCTION

For treatment of castration resistant prostate cancer (CRPC) a systemic chemotherapy has been developed in the last years [1–3]. The chemotherapeutic drug docetaxel (Taxotere®) is given to patients after androgen deprivation therapy (ADT) failure on the basis of improved overall survival, pain reduction, prostate-specific antigen response, and quality of life [3]. However, in many cases its application is limited due to occurrence of an inherent or acquired docetaxel resistance [4]. The survival benefit for CRPC patients is modest being just a few months [5].

It has been hypothesized that development of docetaxel insensitivity is a consequence of a dynamic adaptation of tumor cells to the changing tumor microenvironment during chemotherapy. These adaptations may include but are not limited to protein isoform switching/dysregulation/mutations, alterations in drug efflux mechanisms, and altered expression of pro- and anti-apoptotic proteins [6, 7]. In this context, an acquired docetaxel resistant phenotype has already been associated with changes in isotypes of β-tubulin, the primary target of docetaxel [8, 9], and with multi drug resistance mechanisms (MDRM) including an increased expression of drug transporters like P-glycoprotein [10, 11], or an elevated drug metabolism triggered by high activity of drug detoxifying proteins such as glutathione-S-transferase [12]. In addition, studies have suggested a potential role of anti-apoptotic proteins like members of the inhibitor of apoptosis (IAP) family (XIAP, BIRC5) [13–15] and of the B-cell lymphoma 2 (Bcl-2) family (Bcl-2, Bcl-xL) in chemotherapy resistance [16–18]. However, despite the development of inhibitors against these proteins and their application in clinical trials as single or combination therapies [19–22], the outcome was not satisfying with at best modest results. Thus, identification of new molecular targets is urgently required to combat chemotherapy resistance, improve therapeutic strategies, and prolong patient survival.

Protein inhibitors of activated signal transducer and activator of transcription (STAT) factors (PIAS) proteins, which comprise a family of 4 multifunctional members called PIAS1 to 4, are known to play a role in the modulation of cytokine signaling by inhibiting the activity of STATs [23–25]. PIAS1 and PIAS3 are especially induced by interleukin-6 (IL-6), which was already reported to have an impact on chemotherapy resistance [26, 27]. Besides the DNA and protein binding ability, which is mediated by the conserved SAP domain, PIAS proteins also contain a RING finger-like zinc-binding domain (RLD) as well as a SUMO interaction motif (SIM), thus functioning as SUMO-E3 ligases. Recently, it was demonstrated that PIAS1 mediated SUMOylation is essential for DNA repair [28, 29]. Furthermore, PIAS1 is an important cell cycle regulator, which promotes cell proliferation by SUMOylation triggered inhibition of p73 and p53 [30–32]. As a highly proliferative behavior and suppression of apoptotic stimuli are the main characteristic features of docetaxel resistant cells, the above-mentioned observations render PIAS1 an interesting target protein for further investigation.

In order to address the question if PIAS1 targeting can be used for an improved PCa therapy, we analyzed PIAS1 expression in primary tumors of all stages, in metastatic lesions, in tissue of patients after chemotherapy with docetaxel, and in docetaxel resistant cell lines. Our patient data were complemented by functional experiments after transient and stable PIAS1 knockdown *in vitro* as well as by chick chorioallantoic membrane (CAM) assays and mouse xenograft experiments *in vivo*.

In this study, we confirm elevated PIAS1 expression in PCa and demonstrate for the first time that PIAS1 is, in addition, significantly induced after docetaxel treatment in patients as well as in docetaxel resistant cells *in vitro*. Furthermore, PIAS1 knockdown leads to increased expression of the cell cycle inhibitor p21 and to reduced Mcl1 levels, thereby resulting in induced apoptosis of parental and docetaxel resistant tumor cells.

## RESULTS

### PIAS1 expression is elevated in primary tumors, in metastatic lesions, and in PCa patients after chemotherapy with docetaxel

**Figure 1:**
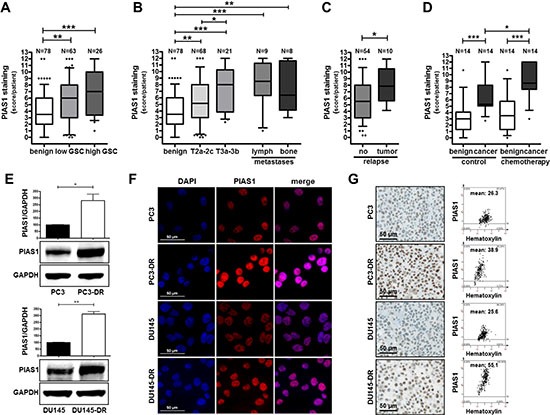
PIAS1 protein expression is elevated in prostate cancer, after docetaxel chemotherapy, in docetaxel resistant cell lines, and in metastases PIAS1 expression was analyzed in PCa tissue samples by immunohistochemistry. Statistical analysis is shown for PIAS1 expression in 78 benign and 89 tumor samples from radical prostatectomy specimens **(A–C)**, as well as in 17 metastatic lesions **(B)**, and in benign and tumor tissue samples from 14 patients who received chemotherapy before radical prostatectomy compared to 14 matched control patients without prior chemotherapy **(D)**. Box-Whiskers plots represent median values, 10–90 percentile (*, *p* < 0.05; **, *p* < 0.01; ***, *p* < 0.001, Mann-Whitney-U-Test). **(E)** PIAS1 protein expression is increased in PC3-DR and DU145-DR cells compared to their parental counterparts. Data represent mean + SD from 3 independent experiments (*, *p* < 0.05; **, *p* < 0.01). Confirmation of elevated PIAS1 protein expression in docetaxel resistant cells by immunofluorescence **(F)** and immunohistochemistry **(G)**. PIAS1 mean intensity was determined by HistoQuest software 4.0, magnification 20x/0.5 DICII, scale bar = 50 μm.

In a previous publication [33] we reported an elevated PIAS1 expression in primary tumors of treatment-naïve PCa patients who had undergone radical prostatectomy. In the present study we extended these findings. Screening of 78 benign and 89 malignant patient samples revealed an induced PIAS1 expression with increasing Gleason score (GSC) [low GSC, ≤7 (3 + 4); high GSC, ≥ 7 (4 + 3)] and tumor stage (Fig. 1A, B; Supplementary Fig. S1A). Our findings in primary PCa samples were strengthened by including metastatic lesions. Immunohistochemical analysis of lymph-node and bone metastases revealed a significant increase in PIAS1 staining compared to benign tissue samples (Fig. 1B; Supplementary Fig. S1A). Moreover, when patients were grouped according to tumor recurrence after ini-tial treatment by radical prostatectomy, a significantly elevated PIAS1 expression was observed in tissues from individuals who experienced biochemical relapse (defined by rising PSA levels) (Fig. 1C). Furthermore, to evaluate the influence of docetaxel on PIAS1 expression, a tissue microarray (TMA) consisting of benign and malignant prostate tissues of 14 PCa patients who received docetaxel before radical prostatectomy, as well as 14 matched treatment-naïve PCa patients [34] was immunohistochemically stained. Semiquantitative analysis revealed significantly increased PIAS1 protein expression in malignant areas compared to corresponding benign samples in both untreated as well as docetaxel treated patient groups (Fig. 1D; Supplementary Fig. S1B). Docetaxel treatment had no influence on PIAS1 expression in benign areas as assessed in a direct comparison between both patient cohorts. Strikingly however, PIAS1 was significantly elevated in malignant areas in the chemotherapy group compared to the control group, which indicates a direct influence of docetaxel treatment on PIAS1 expression selectively in malignant tissues.

### PIAS1 protein expression is increased in docetaxel resistant cells

In order to complement our findings in patient tumor samples we next investigated PIAS1 expression in docetaxel resistant PC3 (PC3-DR) and DU145 (DU145-DR) cells. These cell lines have been established and previously characterized in our laboratory. Western blot analysis revealed significantly increased PIAS1 protein expression in both docetaxel insensitive cell lines (on average 2.5–3 fold increase) compared to their parental counterparts (Fig. 1E). Immunofluorescence and immunohistochemical staining confirmed Western blot results (Fig. 1F, G). Up-regulation of PIAS1 in cells treated with docetaxel seems to be restricted to the development of resistance. Short-term treatment of non-resistant cells with docetaxel caused an inhibitory effect on PIAS1 expression (Supplementary Fig. S2A). Given that PIAS1 itself may be regulated through cell cycle progression [31], the observed effect might be due to the proliferative arrest of parental cells in response to drug treatment. Upregulation of PIAS1 in resistant cells is therefore a long term effect. Consequently, we observed increased levels of NFκB-p100 and two STAT family members, namely STAT3 and STAT5 (Supplementary Fig. S2B) in docetaxel resistant DU145 compared to their parental counterparts, pointing also to a switch in STAT signaling. Taken together, we conclude from these findings that i) PIAS1 is over-expressed in local and metastatic PCa; ii) PIAS1 expression is further induced in prostate tumors after chemotherapeutic treatment with docetaxel; and iii) PCa cells, which survive docetaxel treatment, have significantly elevated PIAS1 levels *in vitro*, thereby suggesting an essential role for PIAS1 during PCa progression and therapy resistance.

### PIAS1 knockdown leads to reduced cell proliferation by p21 up-regulation

**Figure 2:**
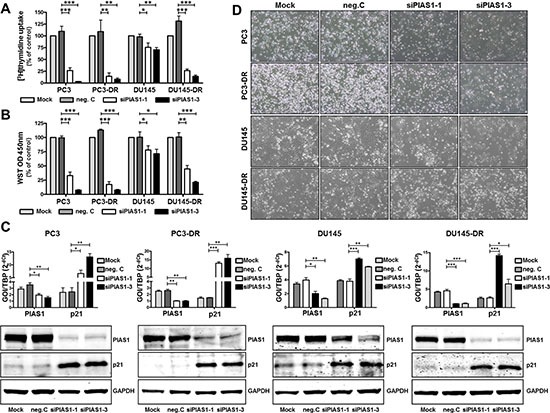
PIAS1 down-regulation leads to reduced cell proliferation through increased expression of cell cycle regulator p21 Cell proliferation in parental and docetaxel resistant cells was assessed after PIAS1 knockdown with 2 specific siRNAs (siPIAS1-1, siPIAS1-3) for 6 days by [^3^H]thymidine incorporation **(A)** and WST assay **(B)**, respectively. Data represent mean + SEM from at least 3 independent experiments (*, *p* < 0.05; **, *p* < 0.01; ***, *p* < 0.001). **(C)** PIAS1 down-regulation and increased p21 expression were assessed at mRNA and protein level by qRT-PCR and Western blot analysis, respectively. Data for qRT-PCR results represent mean + SEM from 3 independent experiments (*, *p* < 0.05; **, *p* < 0.01; ***, *p* < 0.001). **(D)** Reduced cell number of parental and docetaxel resistant cells after specific PIAS1 siRNA treatment.

As our data both from patient material and cell lines revealed increased PIAS1 expression in malignant cells, we next wanted to evaluate the functional significance of PIAS1 and the potential application of PIAS1 knockdown as a new therapy approach. In a previous publication [33] we have already demonstrated that short term PIAS1 down regulation (for 2–4 days) resulted in increased expression of the cell cycle regulator p21 and, in consequence, in decreased proliferation and colony formation ability of PC3, DU145, and VCaP cells. However, under these settings no significant increase in apoptosis was observed. In the present study we were able to confirm and further extend these findings to docetaxel resistant cells. Long term PIAS1 knockdown (for 6 days) using 2 specific PIAS1 siRNAs (siPIAS1-1, siPIAS1-3) resulted in a significant decline in cell proliferation of parental and docetaxel resistant PC3 and DU145 cells, as measured by [^3^H]thymidine uptake and WST assay, respectively (Fig. 2A, B). Reduced cell proliferation was accompanied by elevated p21 levels (Fig 2C). PIAS1 knockdown and p21 expression were controlled in all investigated cell lines at mRNA and protein level by qRT-PCR and Western blot, respectively (Fig. 2C). In addition, treatment with either siRNA resulted in reduced cell numbers after 6 days (Fig. 2D).

### Long term PIAS1 knockdown triggers apoptosis in parental and docetaxel resistant cells *in vitro*

**Figure 3:**
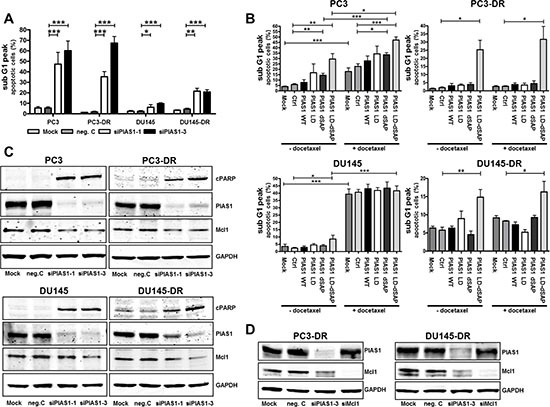
PIAS1 knockdown significantly induces apoptosis *in vitro* **(A)** The proportion of apoptotic cells was measured after 6 days by flow cytometry after PI staining. Data represent mean + SEM from 3 independent experiments (*, *p* < 0.05; **, *p* < 0.01; ***, *p* < 0.001). **(B)** Apoptosis was confirmed by over-expression of PIAS1 mutants in parental as well as in docetaxel resistant cells for 3 days in the absence or presence of docetaxel. Data represent mean + SEM from at least 3 independent experiments (*, *p* < 0.05; **, *p* < 0.01; ***, *p* < 0.001). **(C)** PIAS1 down-regulation results in elevated cPARP and reduced Mcl1 expression as assessed by Western blot. **(D)** PIAS1 knockdown for 3 days leads to reduced Mcl1 protein levels. However, Mcl1 down-regulation has no effect on PIAS1 expression in PC3-DR and DU145-DR cells.

Due to the presence of a considerable number of small and floating cells after treatment with specific PIAS1 siRNAs (Fig. 2D), which indicates increased apoptosis, we verified this hypothesis by measuring the sub G1 fraction by flow cytometry (Fig. 3A). We confirmed an increase in apoptotic cells in both parental as well as docetaxel resistant cell lines after PIAS1 depletion. However, the effect was more pronounced in PC3 and PC3-DR cells. PIAS1 can modify protein activation either by binding to target proteins via the SAP domain, or by promoting SUMOylation via the RLD/SIM motif. To test which domain is crucial for cell survival, we performed transient over-expression of PIAS1 with a wild type (WT) plasmid or PIAS1 constructs harboring either a deletion in the SAP domain (ΔSAP), or a point mutation in the sumo ligase domain (LD), or both (LD-ΔSAP) in the absence or presence of 12.5 nM docetaxel. Over-expression of wild type PIAS1 did not protect PC3 and DU145 cells from docetaxel-induced apoptosis, suggesting that elevated PIAS1 is not a cause of chemotherapy resistance. In contrast to DU145, PC3 cells were sensitized to apoptosis in the presence of both mutant PIAS1 plasmids. However, transfection with a double mutant PIAS1 plasmid (LD-ΔSAP) resulted not only in a significant increase in apoptosis in parental cells, but was also sufficient to induce apoptosis in both docetaxel resistant sub cell lines (Fig. 3B). These results indicate that functional PIAS1 is a critical factor for survival of treatment-naïve and docetaxel resistant cancer cells. Furthermore, transfection with the LD-ΔSAP plasmid and treatment with docetaxel resulted in an additive apoptotic effect in PC3 cells (Fig. 3B).

Elevated apoptosis upon PIAS1 down-regulation was in addition confirmed by increased cPARP levels by Western blot analysis in all investigated cell lines (Fig. 3C). PIAS1 knockdown also reduced expression of the anti-apoptotic protein Mcl1(Fig. 3C). To uncover the hierarchical connection between PIAS1 and Mcl1, we performed siRNA knockdown and subsequent Western blot for both proteins. We observed that PIAS1 knockdown influences Mcl1 expression; Mcl1 depletion, on the other hand, had no influence on PIAS1 levels in docetaxel resistant cells, indicating that PIAS1 is upstream of Mcl1 (Fig. 3D). We have also asked whether PIAS1 downregulation affects expression of other members of the Bcl-2 family and found that expression of neither Bcl-2 nor Bcl-xL is constantly altered in both cell lines following PIAS1 knockdown (Supplementary Fig. S2C). To test whether reduced Mcl1 levels after PIAS1 depletion may be indeed sufficient to induce apoptosis, we measured the percentage of sub-G1 cells after Mcl1 knockdown. Mcl1 depletion caused a significant increase in apoptosis which was similar in parental and resistant cells, however the effect was more pronounced after PIAS1 downregulation (Supplementary Fig. S3A-C).

Collectively, these data suggest that PIAS1 expression is crucial for survival of parental and docetaxel resistant cells, as PIAS1 knockdown results in reduced cell proliferation and elevated apoptosis.

### Mcl1 protein expression is elevated in docetaxel resistant cell lines, in primary PCa tumors, in metastatic lesions, and in patients after docetaxel chemotherapy

**Figure 4:**
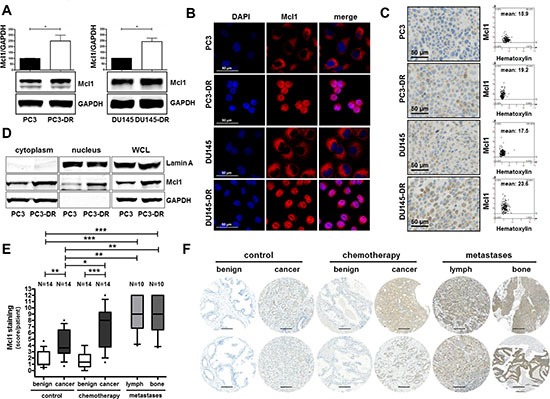
Mcl1 protein expression is elevated in docetaxel resistant cell lines, in prostate cancer, in metastatic lesions, and in patients after docetaxel chemotherapy **(A)** Mcl1 protein expression is increased in PC3-DR and DU145-DR cells compared to their parental counterparts. Data represent mean + SD from 3 independent experiments (*, *p* < 0.05; **, *p* < 0.01). Confirmation of elevated Mcl1 protein expression in docetaxel resistant cells by immunofluorescence **(B)** and immunohistochemistry **(C)**. Mcl1 mean intensity was determined by HistoQuest software 4.0, magnification 20x/0.5 DICII, scale bar = 50 μm. **(D)** Subcellular localization of Mcl1 was assessed by cytoplasmic and nuclear fractionation of PC3 and PC3-DR cell lysates. **(E)** Semi quantitative analysis and **(F)** representative cores of primary tissue samples of untreated or docetaxel treated patients and of metastatic lesions for Mcl1 immunostaining. Box-Whiskers plots represent median values, 10–90 percentile (*, *p* < 0.05; **, *p* < 0.01; ***, *p* < 0.001, Mann-Whitney-*U*-test; magnification 20x/0.5 DICII, scale bar = 100 μm).

Given the observed connection between PIAS1 and Mcl1 as described in Figure 3 and the known important role of Mcl1 during PCa progression due to its anti-apoptotic effects, we investigated Mcl1 expression in docetaxel resistant cells and in tissue of docetaxel treated patients. Western blot analysis revealed significantly elevated Mcl1 expression in PC3-DR and DU145-DR cells (on average 2.5 fold increase) compared to their parental counterparts (Fig. 4A). Immunofluorescence and immunohistochemical staining of all screened cell lines confirmed our Western blot results (Fig. 4B, C). Furthermore, separation of cytoplasmic and nuclear fractions of PC3 and PC3-DR cells revealed increased Mcl1 expression in both cellular compartments of PC3-DR cells (Fig. 4D).

Moreover, semi-quantitative analysis of Mcl1 immunostaining in the TMA, which was also used for PIAS1 immunostaining, revealed significantly increased Mcl1 protein expression in malignant tissue of radical prostatectomy specimens (Fig. 4E). Furthermore, Mcl1 expression was significantly elevated in malignant areas of tumors obtained from patients treated with docetaxel before surgery compared to corresponding benign tissue. Docetaxel treatment had no influence on Mcl1 expression in benign samples. Strikingly however, Mcl1 protein was significantly induced in malignant areas of the chemotherapy group compared to the control group (Fig. 4E, F). Finally, immunohistochemical analysis of lymph node and bone metastases revealed significantly increased Mcl1 staining in metastatic lesions compared to benign samples (Fig. 4E, F). Hence, we conclude that Mcl1 protein, similar to PIAS1, is over-expressed in primary and metastatic PCa and is also further elevated after docetaxel treatment.

### PIAS1 knockdown influences tumor growth of PC3 and PC3-DR CAM onplants

Having shown that PIAS1 knockdown results in increased apoptosis *in vitro*, we next aimed to confirm our findings *in vivo*. We therefore investigated consequences of PIAS1 down-regulation on PC3 and PC3-DR tumor growth in a CAM assay. For this purpose, we established PC3 and PC3-DR sub lines containing a doxycycline inducible GFP tagged shRNA vector targeting PIAS1 (shPIAS1-1, shPIAS1-3) or a control vector containing shRNA against luciferase (shLuc). Possible toxic side effects of doxycycline were excluded in control experiments. Doxycycline up to a concentration of 4 μg/ml had no influence on cell proliferation in PC3shLuc and PC3-DRshLuc cells *in vitro* (Supplementary Fig. S4). Both PIAS1 shRNA sequences significantly reduced PIAS1 protein expression in PC3 and PC3-DR sub cell lines which resulted in diminished cell proliferation. However, the shPIAS1-3 sequence had a more pronounced anti-proliferative effect in both tested cell lines. Activation of the inducible system with 1 μg/ml doxycycline was sufficient to reduce PIAS1 expression and, in consequence, proliferation (Supplementary Fig. S5A-D).

**Figure 5:**
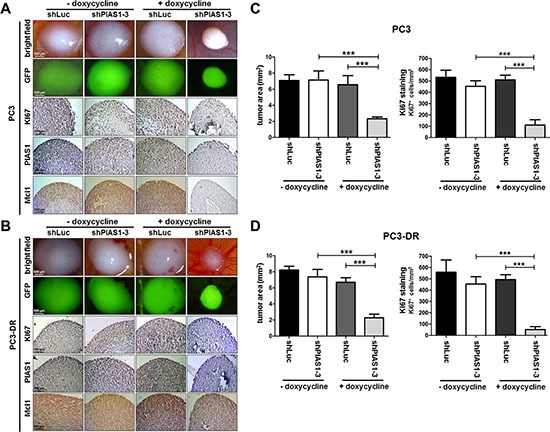
PIAS1 knockdown influences proliferation and tumor growth of PC3 and PC3-DR CAM onplants *in vivo* **(A–B)** Representative brightfield- and fluorescence images of whole tumors as well as representative pictures taken after immunohistochemical Ki67, PIAS1, and Mcl1 staining of tumor cross sections. **(C, D)** Statistical analysis of tumor area and Ki67 positive cells (cells/cm^2^ tumor cross-section) of PC3 and PC3-DR tumors. Data represent mean + SD of 5 onplants/treatment done in 2 independent experiments (***, *p* < 0.001).

Strikingly, PIAS1 knockdown for 6 days using the shPIAS1-3 sequence and 1 μg/ml doxycycline resulted in a significant reduction in cell proliferation and tumor volume of PC3 (Fig. 5A, C) and PC3-DR (Fig. 5B, D) onplants in the CAM experiment. These findings were confirmed by a significantly reduced number of Ki67 positive cells in all shPIAS1-3 onplants and by reduced PIAS1, Ki67, and Mcl1 immunoreactivity in PC3 as well as in PC3-DR cells of the specific shPIAS1-3 treatment group (Fig. 5A, B).

### PIAS1 knockdown results in reduced tumor growth of PC3 and PC3-DR mouse xenografts

**Figure 6:**
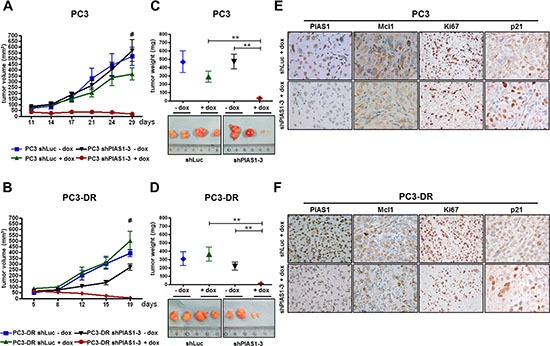
PIAS1 knockdown results in significantly reduced tumor growth of PC3 and PC3-DR xenografts *in vivo* Specific PIAS1 knockdown through activation of the doxycline-inducible system leads to a significant reduction in tumor volume **(A, B)** and tumor weight **(C, D)** in the shPIAS1-3+dox treatment group compared to the control groups (*, *p* < 0.05; **, *p* < 0.01; ***, *p* < 0.001; mean + SEM). Shown are 2 representative tumors for each group. **(E, F)** Representative pictures for PIAS1, Mcl1, Ki67, and p21 IHC-staining of tumors from the shLuc+dox and shPIAS1-3+dox treatment group (magnification 400x).

So far, we were able to demonstrate increased PIAS1 levels in docetaxel treated patients and in docetaxel resistant cell lines and proved that PIAS1 expression is crucial for cancer cell survival. Finally, we translated these findings in a mouse xenograft model. For this purpose, we used PC3 and PC3-DR sub lines stably transfected with shPIAS1-3 or shLuc as described above. For the PC3 as well as for the PC3-DR xenograft experiments, the same number of mice was randomly divided into four treatment groups; shLuc-dox, shLuc+dox, shPIAS1-3-dox, and shPIAS1-3+dox. After tumors had established, shPIAS1-3 or shLuc expression was induced by adding doxycycline into the drinking water of the respective +dox groups. All tumors in the 3 control groups constantly gained volume over time. Strikingly however, PIAS1 knockdown in the shPIAS1-3+dox group resulted in a complete abrogation of tumor growth in both PC3 and PC3-DR xenografts and was furthermore even sufficient to induce partial tumor regression (Fig. 6A, B). Even more, 3 out of 7 mice in the PC3 xenograft and 5 out of 7 mice in the PC3-DR xenograft had no detectable tumor mass at the end of the study, demonstrating complete tumor regression upon PIAS1 knockdown. Determination of tumor volume and weight at the end of the experiment revealed a significant decrease in both parameters in tumors that arose from cells where PIAS1 was depleted (Figure 6C, D). Subsequent immunohistochemical staining of tumors confirmed PIAS1 knockdown in the specific treatment group and furthermore revealed decreased Mcl1, Ki67 and elevated p21 expression compared to tumors of the control groups (Fig. 6E, F; Supplementary Fig. S6).

## DISCUSSION

PIAS proteins are important regulators of many cellular pathways by influencing the activity and stability of target proteins through their SAP domain or SUMO E3 ligase activity. Therefore, a well-balanced expression of PIAS proteins is critical for normal cell homeostasis and their deregulation might be one reason for cancer initiation or progression. However, the expression status and the functional role of PIAS proteins in cancer, including PCa, have not been investigated in detail yet. So far, Brantley and colleagues could demonstrate that loss of PIAS3 leads to enhanced proliferation in glioblastoma multiforme, while Coppola et al. associated reduced expression of PIAS1 with colon cancer development [35, 36]. In contrast to these findings, we observed elevated PIAS1 expression in PCa and proposed a pro-proliferative role for the protein in this malignancy, as PIAS1 was co-expressed with the proliferation markers Ki67 and PCNA in PCa tissue [33]. Our observations were supported by findings of Li and colleagues, who reported increased PIAS1 mRNA levels in PCa samples [37]. Moreover, it was demonstrated that PIAS1 is a co-activator of the androgen receptor (AR) and its mRNA expression is up-regulated by androgenic hormones [38, 39]. Thus, PIAS1 may in addition contribute to enhanced proliferation or decreased apoptosis of PCa cells through stimulation of AR activity in this malignancy. In future studies, it may be of interest to evaluate the role of PIAS1 in AR-positive docetaxel resistant PCa cells. However, the attempts from the authors´ laboratory to generate such a subline *in vitro* have not been successful so far.

To further extend our knowledge on PIAS1 and to evaluate if PIAS1 targeting can improve current cancer therapies, we performed a detailed analysis of PIAS1 expression and function in PCa. In our comprehensive expression studies we evaluated PIAS1 levels in non-cancerous prostate tissues, primary tumors of different grades and stages, metastatic lesions, and chemotherapy-treated tumor specimens (217 tissue samples in total), as well as in parental and docetaxel resistant PCa cell lines. In summary, tissue data presented in this manuscript confirm that PIAS1 is over-expressed in local and metastatic PCa and is, in addition, elevated in patients with biochemical recurence after radical prostatectomy. Moreover, we have proven for the first time that PIAS1 is even further induced in tissue of docetaxel treated versus untreated patients as well as in docetaxel resistant cell lines. We thus hypothesize that chemotherapeutic treatment with docetaxel leads to a clonal selection of highly proliferative cells with pronounced PIAS1 expression. This assumption is supported by the fact that we already have demonstrated a docetaxel-induced clonal selection of highly proliferative and invasive docetaxel resistant cancer cells that display a mesenchymal phenotype and harbor stem cell-like properties [34]. Strikingly, functional data of our *in vitro* and *in vivo* studies identify PIAS1 as a crucial factor for tumor cell survival since PIAS1 knockdown resulted in reduced proliferation and tumor growth as well as increased apoptosis in parental and in docetaxel resistant cells.

PIAS1 was originally identified as an inhibitor of STAT1. It is well known that activated STAT factors can regulate gene expression and thereby influence cell differentiation, proliferation, angiogenesis, and apoptosis. In stress-induced responses they are activated by cytokine signaling and modulate pro- and anti-apoptotic genes. STAT1 was initially thought to be a tumor suppressor as STAT1-deficient mice developed tumors and STAT1-deficient cancer cells were found to be more resistant to chemotherapy [40]. However, elevated STAT1 expression was also associated with chemotherapy resistance in PCa cells. Patterson and colleagues observed an increased STAT1 expression in docetaxel resistant DU145 cells and concluded that high STAT1 levels in combination with elevated clusterin expression are essential for docetaxel resistance [41]. Partly, we were able to confirm these findings, given that we also observed increased STAT1 and clusterin levels in our own developed docetaxel resistant PC3 cells [34].

However, the mechanistic background as well as functional consequences of altered STAT1 levels have not been investigated so far. Based on our data we hypothesize that elevated PIAS1 expression in docetaxel resistant cells impairs transcriptional activity of STAT1 via inhibition of its DNA binding ability. Therefore, these cells might activate compensatory mechanisms, leading to increased transcription of STAT1 and other STAT factors such as STAT3 and STAT5 and in consequence to the upregulation of anti-apoptotic proteins, e.g. Mcl1, survivin or c-fos. Indeed, in this study we found increased levels of STAT3 and STAT5 in DU145-DR compared to parental cells. However, PC3 as well as PC3-DR cells are negative for both proteins. Therefore, additional functional experiments are warranted, including evaluation of STAT1 phosphorylation at different sites and the interaction with other STAT family members to uncover the specific role of STAT factors in chemotherapy resistance. Additionally, one has to keep in mind that PIAS1 functions are not limited to STAT factors.

PIAS1 already has been identified as a negative regulator of tumor suppressors such as p53 and p73 [30–32]. Both proteins negatively influence cell proliferation by directly activating cell cycle inhibitor p21 [31, 42]. In this context, we confirmed and were able to extend our previous *in vitro* findings [33] by demonstrating that transient and stable long term PIAS1 knockdown (≥6 days) causes increased expression of p21 and, in addition, a significant repression of the Bcl-2 family member Mcl1. In consequence, this leads to reduced proliferation and sensitization of parental and, importantly, docetaxel resistant tumor cells to apoptosis. Interestingly, over-expression of wild-type or mutant PIAS1 revealed that the SAP- as well as the SUMO ligase domain have to be inactive in order to successfully initiate apoptosis. This fact has to be considered in the development of future PIAS1 inhibitors.

As Mcl1 seems to be influenced by PIAS1 we also evaluated Mcl1 expression in docetaxel resistant cell lines, in metastatic lesions, as well as in chemotherapy-naïve and treated patients. Hence, we could not only demonstrate that Mcl1 expression is elevated in primary and metastatic tumors which confirms previous observations by Krajewska et al. and Zhang et al. [43, 44], but were also able to demonstrate that its expression, similar to that of PIAS1, is further induced after docetaxel chemotherapy and in resistant cells. *In vitro*, elevated Mcl1 levels have already been implicated in resistance to cytokine-induced apoptosis and may also be involved in the anti-apoptotic action of IL-6 [45], which was also associated with chemotherapy resistance [26, 27]. Previously developed multi target inhibitors, like AT-101, which targets several Bcl-2 family members including Mcl1, have been tested in clinical studies, however, with modest results. In a randomized phase I/II trial including 23 men with chemotherapy- naïve CRPC, the administration of AT-101 (20 mg/day for 21 of 28 days) resulted in reduced PSA levels in some patients [46]. Nevertheless, in another randomized, double-blind, placebo-controlled phase II trial including 221 men with progressive CRPC, treatment with AT-101 in combination with docetaxel and prednisone did not result in extended overall survival compared to standard treatment plus placebo [47]. In this context, we speculate that additional PIAS1 knockdown to standard treatment could improve therapeutic outcome of CRPC patients as down-regulation of PIAS1 in combination with docetaxel treatment resulted in an additive apoptotic effect in PC3 cells *in vitro*. However, a potential benefit of such a combined treatment approach has to be evaluated employing additional cell lines as well as *in vivo* models.

Taken together, our findings confirm that PIAS1 is over-expressed in PCa and show for the first time that PIAS1 expression is significantly increased in docetaxel resistant cells *in vitro* and in tissue of patients after chemotherapy with docetaxel. Furthermore, we demonstrate that PIAS1 is a crucial factor for survival of treatment-naïve and docetaxel resistant prostate cancer cells. PIAS1 down-regulation causes elevated p21 and reduced Mcl1 levels, which consequently results in increased cell death. On the basis of the present data, we conclude that PIAS1 may be a promising new target for treatment of primary, metastatic, and chemotherapy resistant PCa.

## METHODS

### Ethics statement

Investigation has been conducted in accordance with the ethical standards and according to the Declaration of Helsinki and according to national and international guidelines and has been approved by the authors' institutional review board.

### Cell culture and chemicals

PC3 and DU145 cells were obtained from the American Type Culture Collection (ATCC, Rockville, MD). Docetaxel resistant cell lines PC3-DR and DU145-DR were cultured as described previously [34]. Cell lines were cultured in RPMI 1640 supplemented with 10% fetal calf serum (FCS) and 20 mM glutamine. Representative bright-field images of cells were taken using IC Capture Software 2.2 with an Olympus CK2 microscope (Olympus, Vienna, Austria) equipped with an Imaging Source Camera DFK31F03 (Imaging Source, Bremen, Germany). Identity of the used cell lines was confirmed by short tandem repeat analysis.

### Tissue microarray (TMA) and immunohistochemistry

Evaluation of PIAS1 expression was performed in 78 benign and 89 malignant tissues of PCa patients. To investigate potential changes in PIAS1 and Mcl1 expression following chemotherapy, a TMA of formalin-fixed, paraffin-embedded tissue blocks of 14 PCa patients who underwent neoadjuvant chemotherapy with docetaxel before radical prostatectomy, as well as 14 matched of treatment-naïve radical prostatectomy PCa patients was employed as described in detail elsewhere [34]. In addition, TMAs comprising bone and lymph-node metastases from 10 patients were stained for PIAS1 and Mcl1 expression. The use of archived material was approved by the Ethics Committee of Innsbruck Medical University (Study no. AM 3174 including amendment 2). Immunohistochemistry for PIAS1 and Mcl1 was performed on a Discovery - XT staining device (Ventana, Tucson, AZ). The following antibodies were used: anti-PIAS1 (1:400; Abcam, Cambridge, UK) and anti-Mcl1 (1:200; Santa Cruz Biotechnology, Santa Cruz, CA). Additionally, formalin-fixed and paraffin-embedded PC3, DU145, PC3-DR, and DU145-DR were stained with anti-PIAS1 (1:400; Abcam) and anti-Mcl1 (1:200; Santa Cruz). Images were taken with a Zeiss Imager Z2 microscope (Zeiss, Vienna) equipped with a Pixelink PL-B622-CU camera (Canimpex Enterprises Ltd., Halifax, Canada). Immunohistochemical evaluation was done by an uropathologist (G.S.) using the semi-quantitative scoring system “quick score” combining the proportion of positive cells and the average staining intensity. PIAS1 and Mcl1 expression in PC3, DU145, PC3-DR, and DU145-DR cells was quantified using the HistoQuest 3.0 software (Tissue Gnostics, Vienna).

### Immunofluorescence

Cells were seeded onto glass coverslips and treated as previously described [33]. Coverslips were incubated with primary antibodies against PIAS1 (1:50; Cell Signaling) and Mcl1 (1:100; Santa Cruz) for 1 h. After washing, coverslips were incubated with the fluorescence-labeled secondary antibody goat anti-rabbit 555 (Invitrogen, Carlsbad, CA). Coverslips were finally washed and mounted with Vectashield Hard Set mounting medium containing DAPI (Vector Laboratories, Burlingame, CA). The cells were visualized using fluorescence microscopy on a Zeiss Axio Imager Z2 microscope (Zeiss, Vienna).

### Short interfering RNA (siRNA) transfection

The following short interfering RNA sequences were used for targeting human PIAS1 and Mcl1: siPIAS1-1, 5′-AAGGUCAUUCUAGAGCUUUAdTdT-3′, siPIAS1-3 5′-CGAAUGAACUUGGCAGAAAdTdT-3′, and siMcl1 5′-GCAAGUGGCAAGAGGAUUAdtdt-3′. A non targeting siRNA pool [Cat.nr.: D-001810-10-20, Dharmacon, (Chicago, IL)] was used as a negative control. siRNA transfections were performed with Lipofectamine 2000 (Invitrogen) reagent according to the manufacturer's protocol. All cell lines were transfected with either 25 nM of siRNAs against PIAS1, Mcl1, or non-targeting control. To ensure sustained down-regulation of the target proteins for 6 days, all cell lines were re-transfected with the respective siRNA at day 3.

### PIAS1 plasmids

Expression vectors pEGFP-C1-PIAS1-WT, pEGFP-C1-PIAS1-LD, pEGFP-C1-PIAS1-ΔSAP, and pEGFP-C1-PIAS1-LDΔSAP were generated by Dr. Yaron Galanty (Gurdon Institute, Cambridge) as described elsewhere [29]. Cells were transfected with 3 μg/ml of DNA using Fugene HD transfection reagent (Roche, Basel, Switzerland) for 72 h following the manufacturer's instructions.

### qRT-PCR

Total RNA was isolated using the RNeasy mini kit (Qiagen, Hilden, Germany) and cDNA synthesis was performed using cDNA RT^2^ first strand kit (Qiagen). qRT-PCR was performed as described elsewhere [48]. TATA-Box binding protein (TBP) was chosen as an endogenous expression standard (forward 5′-CACGAACCACGGCACTGATT-3′; reverse 5′-TTTTCTGCTGCCAGTCTGGAC-3′; probe 5′-FAM-TCTTCACTCTTGGCTCCTGTGCACA-TAMRA-3′). For PIAS1, p21, and Mcl1, Taqman gene expression assays, purchased from Applied Biosystems, were used according to the manufacturer's protocol.

### Western blot

Western blot was performed as previously described [48]. The following antibodies were used: anti-GAPDH (1:100000; Chemicon, Vienna), anti-Mcl1 (1:500; Santa Cruz), anti-cPARP (1:1000; Promega, Madison, WI), anti-PIAS1 (1:500; Cell Signaling, Danvers, MA), anti-p21^CIP1/WAF1^ (1:500; Cell Signaling), anti-p100 (1:500; Cell Signaling), anti-STAT3 (1:1000; Santa Cruz), anti-STAT5, (1:1000; Santa Cruz), anti-Bcl-2 (1:500; Cell Signaling), and anti-Bcl-xL (1:500; Cell Signaling).

### [^3^H]thymidine incorporation, WST assay, and apoptosis measurement

For [^3^H]thymidine incorporation and WST assays, cells were seeded at a density of 2.5 × 10^3^/well in triplicates onto separate 96-well plates. For apoptosis measurement, cells were seeded at a density of 1.5 × 10^5^/well onto 6-well plates. Cells were transfected twice in a period of 6 days. Doxycycline inducible sub cell lines were cultured in the presence or absence of doxycycline. Thymidine incorporation was measured as previously described [48]. As an index of cell proliferation and viability a WST assay (Roche) was performed according to the manufacturer's protocol. The percentage of apoptotic cells was assessed as previously described [33].

### Establishment of doxycycline inducible shRNA constructs

PC3 and PC3-DR cells were stably infected with doxycycline-inducible shRNA contructs against PIAS1 or luciferase [(shPIAS1-1, 5′-_GATCCCC_AAGGTCATTCTAGAGCTTTA_TTCAAGAGA_TAAAGCTCTAGAATGACCTT_TTTTTGGAAA_-3′; shPIAS1-3, 5′-_GATCCCC_CGAATGAACTTGGCAGAAA_TTCAAGAGA_TTTCTGCCAAGTTCATTCG_TTTTTGGAAA_-3′ or shLuc, 5′-_GATCCCC_CTTACGCTGAGTACTTCGA_TTCAAGAGA_TCGAAGTACTCAGCGTAAG_TTTTTGGAAA_-3′]. Generation and transfection of constructs were performed as previously described [49].

### CAM assay

CAM assay was performed as described elsewhere [50] with slight modifications. For onplant preparation, native, non-pepsinized type I rat tail collagen (BD Bioscience, Bedford, MA) was neutralized with 0.2 M NaOH solution and mixed with 10 × DMEM medium. 5 × 10^5^ PC3 or PC3-DR cells were added to 50 μL of this solution. Collagen-onplants with or without doxycyclin (1 μg/ml) were applied to CAMs and incubated for 5 days. Xenografts were analyzed under a stereomicroscope with a digital camera (Olympus SZX10, Olympus E410, Vienna). For histological analysis, onplants were excised from the CAM, fixed in 4% paraformaldehyde, and processed for paraffin sectioning and IHC. The following antibodies were used: anti-PIAS1 (1:400; Abcam), anti-Mcl1 (1:200; Santa Cruz), and anti-Ki67 (1:100; DAKO, Glostrup, Denmark).

### Establishment and treatment of human prostate tumor xenografts in nude mice

Animal protocols were approved by the Austrian Federal Ministry of Science (BMWF-66.011/0099-II/3b/2012). All efforts were made to minimize suffering of the animals. 4–6 weeks old male nude mice (BALB/c/*nu*/*nu*) were purchased from Charles River Laboratories (Sulzfeld, Germany) and were housed under pathogen-free conditions. Xenograft tumors were grown by subcutaneous implantation of a 100 μl (1:1) suspension of 2 × 10^6^ PC3-shLuc and PC3-shPIAS1-3 cells or 1.5 × 10^6^ PC3-DR-shLuc and PC3-DR-shPIAS1-3 cells mixed with matrigel (BD Biosciences) into the right and the left flanks of mice, respectively. For the PC3 as well as for the PC3-DR xenograft experiment, the same amount of mice was randomly divided into four treatment groups: shLuc-dox (N = 5), shluc+dox (N = 5), shPIAS1-3-dox (N = 5), and shPIAS1-3+dox (N = 7). Doxycycline was administered at a concentration of 1g/l via drinking water after tumors reached the volume of 50 mm^3^. Water bottles were changed 3 times a week. Tumor sizes were determined by caliper measurements and calculated with the formula Volume = (width)^2^ × length/2 at least once a week. Each tumor was measured individually. After mice were sacrificed, tumors were fixed in buffered formalin (4.5%) and embedded in paraffin for further immunohistochemical staining. The following antibodies were used: anti-PIAS1 (1:400; Abcam), anti-Mcl1 (1:200; Santa Cruz), anti-Ki67 (1:100; DAKO), and anti-p21 (1:100; Abcam).

### Statistical analysis

SPSS (V15.0) and GraphPad Prism 5 were used for statistical analyses. For all experiments, Gaussian distribution was determined using Kolmogorov-Smirnov test. Differences between treatment groups were analyzed using Student's *t*-test or Mann–Whitney *U*-test. *P* values below 0.05 were considered significant. Tumor volume/time was corrected for multiple testing using Bonferroni method in all *in vivo* xenograft experiments. All differences highlighted by asterisks were statistically significant as encoded in figure legends (*P < 0.05, **P < 0.01, ***P < 0.001). Data are presented as mean + SD unless otherwise specified.

## SUPPLEMENTARY FIGURES



## References

[1] Saad F, Ruether D, Ernst S, North S, Cheng T, Perrotte P, Karakiewicz P, Winquist E (2008). The Canadian Uro-Oncology Group multicentre phase II study of docetaxel administered every 3 weeks with prednisone in men with metastatic hormone-refractory prostate cancer progressing after mitoxantrone/prednisone. BJU international.

[2] Tannock IF, de Wit R, Berry WR, Horti J, Pluzanska A, Chi KN, Oudard S, Theodore C, James ND, Turesson I, Rosenthal MA, Eisenberger MA (2004). Docetaxel plus prednisone or mitoxantrone plus prednisone for advanced prostate cancer. The New England journal of medicine.

[3] Petrylak DP, Tangen CM, Hussain MH, Lara PN, Jones JA, Taplin ME, Burch PA, Berry D, Moinpour C, Kohli M, Benson MC, Small EJ, Raghavan D, Crawford ED (2004). Docetaxel and estramustine compared with mitoxantrone and prednisone for advanced refractory prostate cancer. The New England journal of medicine.

[4] Geney R, Ungureanu lM, Li D, Ojima I (2002). Overcoming multidrug resistance in taxane chemotherapy. Clinical chemistry and laboratory medicine : CCLM / FESCC.

[5] Armstrong AJ, Garrett-Mayer E, de Wit R, Tannock I, Eisenberger M (2010). Prediction of survival following first-line chemotherapy in men with castration-resistant metastatic prostate cancer. Clin Cancer Res.

[6] Murray S, Briasoulis E, Linardou H, Bafaloukos D, Papadi-mitriou C (2012). Taxane resistance in breast cancer: mechanisms, predictive biomarkers and circumvention strategies. Cancer Treat Rev.

[7] Mahon KL, Henshall SM, Sutherland RL, Horvath LG (2011). Pathways of chemotherapy resistance in castration-resistant prostate cancer. Endocr Relat Cancer.

[8] Ploussard G, Terry S, Maille P, Allory Y, Sirab N, Kheuang L, Soyeux P, Nicolaiew N, Coppolani E, Paule B, Salomon L, Culine S, Buttyan R, Vacherot F, de la Taille A (2010). Class III beta-tubulin expression predicts prostate tumor aggressiveness and patient response to docetaxel-based chemotherapy. Cancer Res.

[9] Terry S, Ploussard G, Allory Y, Nicolaiew N, Boissiere-Michot F, Maille P, Kheuang L, Coppolani E, Ali A, Bibeau F, Culine S, Buttyan R, de la Taille A, Vacherot F (2009). Increased expression of class III beta-tubulin in castration-resistant human prostate cancer. Br J Cancer.

[10] Sanchez C, Mendoza P, Contreras HR, Vergara J, McCubrey JA, Huidobro C, Castellon EA (2009). Expression of multidrug resistance proteins in prostate cancer is related with cell sensitivity to chemotherapeutic drugs. Prostate.

[11] Lee JT, Steelman LS, McCubrey JA (2004). Phosphatidylinositol 3′-kinase activation leads to multidrug resistance protein-1 expression and subsequent chemoresistance in advanced prostate cancer cells. Cancer Res.

[12] Arai T, Miyoshi Y, Kim SJ, Akazawa K, Maruyama N, Taguchi T, Tamaki Y, Noguchi S (2008). Association of GSTP1 expression with resistance to docetaxel and paclitaxel in human breast cancers. Eur J Surg Oncol.

[13] Rahman KM, Banerjee S, Ali S, Ahmad A, Wang Z, Kong D, Sakr WA (2009). 3,3′-Diindolylmethane enhances taxotere-induced apoptosis in hormone-refractory prostate cancer cells through survivin down-regulation. Cancer Res.

[14] Ling X, Cao S, Cheng Q, Keefe JT, Rustum YM, Li F (2012). A novel small molecule FL118 that selectively inhibits survivin, Mcl-1, XIAP, and cIAP2 in a p53-independent manner, shows superior antitumor activity. PLoS One.

[15] Hayashi N, Asano K, Suzuki H, Yamamoto T, Tanigawa N, Egawa S, Manome Y (2005). Adenoviral infection of survivin antisense sensitizes prostate cancer cells to etoposide in vivo. Prostate.

[16] Yoshino T, Shiina H, Urakami S, Kikuno N, Yoneda T, Shigeno K, Igawa M (2006). Bcl-2 expression as a predictive marker of hormone-refractory prostate cancer treated with taxane-based chemotherapy. Clin Cancer Res.

[17] Vilenchik M, Raffo AJ, Benimetskaya L, Shames D, Stein CA (2002). Antisense RNA down-regulation of bcl-xL Expression in prostate cancer cells leads to diminished rates of cellular proliferation and resistance to cytotoxic chemotherapeutic agents. Cancer Res.

[18] Sjostrom J, Blomqvist C, von Boguslawski K, Bengtsson NO, Mjaaland I, Malmstrom P, Ostenstadt B, Wist E, Valvere V, Takayama S, Reed JC, Saksela E (2002). The predictive value of bcl-2, bax, bcl-xL, bag-1, fas, and fasL for chemotherapy response in advanced breast cancer. Clin Cancer Res.

[19] Morris MJ, Cordon-Cardo C, Kelly WK, Slovin SF, Siedlecki K, Regan KP, DiPaola RS, Rafi M, Rosen N, Scher HI (2005). Safety and biologic activity of intravenous BCL-2 antisense oligonucleotide (G3139) and taxane chemotherapy in patients with advanced cancer. Appl Immunohistochem Mol Morphol.

[20] Tolcher AW, Kuhn J, Schwartz G, Patnaik A, Hammond LA, Thompson I, Fingert H, Bushnell D, Malik S, Kreisberg J, Izbicka E, Smetzer L, Rowinsky EK (2004). A Phase I pharmacokinetic and biological correlative study of oblimersen sodium (genasense, g3139), an antisense oligonucleotide to the bcl-2 mRNA, and of docetaxel in patients with hormone-refractory prostate cancer. Clin Cancer Res.

[21] Satoh T, Okamoto I, Miyazaki M, Morinaga R, Tsuya A, Hasegawa Y, Terashima M, Ueda S, Fukuoka M, Ariyoshi Y, Saito T, Masuda N, Watanabe H, Taguchi T, Kakihara T, Aoyama Y (2009). Phase I study of YM155, a novel survivin suppressant, in patients with advanced solid tumors. Clin Cancer Res.

[22] Dean E, Jodrell D, Connolly K, Danson S, Jolivet J, Durkin J, Morris S, Jowle D, Ward T, Cummings J, Dickinson G, Aarons L, Lacasse E, Robson L, Dive C, Ranson M (2009). Phase I trial of AEG35156 administered as a 7-day and 3-day continuous intravenous infusion in patients with advanced refractory cancer. J Clin Oncol.

[23] Sharrocks AD (2006). PIAS proteins and transcriptional regulation–more than just SUMO E3 ligases?. Genes Dev.

[24] Liu B, Liao J, Rao X, Kushner SA, Chung CD, Chang DD, Shuai K (1998). Inhibition of Stat1-mediated gene activation by PIAS1. Proc Natl Acad Sci USA.

[25] Chung CD, Liao J, Liu B, Rao X, Jay P, Berta P, Shuai K (1997). Specific inhibition of Stat3 signal transduction by PIAS3. Science.

[26] Domingo-Domenech J, Oliva C, Rovira A, Codony-Servat J, Bosch M, Filella X, Montagut C, Tapia M, Campas C, Dang L, Rolfe M, Ross JS, Gascon P, Albanell J, Mellado B (2006). Interleukin 6, a nuclear factor-kappaB target, predicts resistance to docetaxel in hormone-independent prostate cancer and nuclear factor-kappaB inhibition by PS-1145 enhances docetaxel antitumor activity. Clin Cancer Res.

[27] Codony-Servat J, Marin-Aguilera M, Visa L, Garcia-Albeniz X, Pineda E, Fernandez PL, Filella X, Gascon P, Mellado B (2013). Nuclear factor-kappaB and interleukin-6 related docetaxel resistance in castration-resistant prostate cancer. Prostate.

[28] Shima H, Suzuki H, Sun J, Kono K, Shi L, Kinomura A, Horikoshi Y, Ikura T, Ikura M, Kanaar R, Igarashi K, Saitoh H, Kurumizaka H, Tashiro S (2013). Activation of the SUMO modification system is required for the accumulation of RAD51 at sites of DNA damage. J Cell Sci.

[29] Galanty Y, Belotserkovskaya R, Coates J, Polo S, Miller KM, Jackson SP (2009). Mammalian SUMO E3-ligases PIAS1 and PIAS4 promote responses to DNA double-strand breaks. Nature.

[30] Kahyo T, Nishida T, Yasuda H (2001). Involvement of PIAS1 in the sumoylation of tumor suppressor p53. Mol Cell.

[31] Munarriz E, Barcaroli D, Stephanou A, Townsend PA, Maisse C, Terrinoni A, Neale MH, Martin SJ, Latchman DS, Knight RA, Melino G, De Laurenzi V (2004). PIAS-1 is a checkpoint regulator which affects exit from G1 and G2 by sumoylation of p73. Mol Cell Biol.

[32] Schmidt D, Muller S (2002). Members of the PIAS family act as SUMO ligases for c-Jun and p53 and repress p53 activity. Proc Natl Acad Sci USA.

[33] Hoefer J, Schafer G, Klocker H, Erb HH, Mills IG, Hengst L, Puhr M, Culig Z (2012). PIAS1 is increased in human prostate cancer and enhances proliferation through inhibition of p21. Am J Pathol.

[34] Puhr M, Hoefer J, Schafer G, Erb HH, Oh SJ, Klocker H, Heidegger I, Neuwirt H, Culig Z (2012). Epithelial-to-mesenchymal transition leads to docetaxel resistance in prostate cancer and is mediated by reduced expression of miR-200c and miR-205. Am J Pathol.

[35] Coppola D, Parikh V, Boulware D, Blanck G (2009). Substantially reduced expression of PIAS1 is associated with colon cancer development. J Cancer Res Clin Oncol.

[36] Brantley EC, Nabors LB, Gillespie GY, Choi YH, Palmer CA, Harrison K, Roarty K, Benveniste EN (2008). Loss of protein inhibitors of activated STAT-3 expression in glioblastoma multiforme tumors: implications for STAT-3 activation and gene expression. Clin Cancer Res.

[37] Li P, Yu X, Ge K, Melamed J, Roeder RG, Wang Z (2002). Heterogeneous expression and functions of androgen receptor co-factors in primary prostate cancer. Am J Pathol.

[38] Gross M, Liu B, Tan J, French FS, Carey M, Shuai K (2001). Distinct effects of PIAS proteins on androgen-mediated gene activation in prostate cancer cells. Oncogene.

[39] Heemers HV, Regan KM, Schmidt LJ, Anderson SK, Ballman KV, Tindall DJ (2009). Androgen modulation of coregulator expression in prostate cancer cells. Mol Endocrinol.

[40] Stephanou A, Latchman DS (2003). STAT-1: a novel regulator of apoptosis. Int J Exp Pathol.

[41] Patterson SG, Wei S, Chen X, Sallman DA, Gilvary DL, Zhong B, Pow-Sang J, Yeatman T, Djeu JY (2006). Novel role of Stat1 in the development of docetaxel resistance in prostate tumor cells. Oncogene.

[42] Nozell S, Chen X (2002). p21B, a variant of p21(Waf1/Cip1), is induced by the p53 family. Oncogene.

[43] Krajewska M, Krajewski S, Epstein JI, Shabaik A, Sauvageot J, Song K, Kitada S, Reed JC (1996). Immunohistochemical analysis of bcl-2, bax, bcl-X, and mcl-1 expression in prostate cancers. Am J Pathol.

[44] Zhang S, Zhau HE, Osunkoya AO, Iqbal S, Yang X, Fan S, Chen Z, Wang R, Marshall FF, Chung LW, Wu D (2010). Vascular endothelial growth factor regulates myeloid cell leukemia-1 expression through neuropilin-1-dependent activation of c-MET signaling in human prostate cancer cells. Mol Cancer.

[45] Cavarretta IT, Neuwirt H, Untergasser G, Moser PL, Zaki MH, Steiner H, Rumpold H, Fuchs D, Hobisch A, Nemeth JA, Culig Z (2007). The antiapoptotic effect of IL-6 autocrine loop in a cellular model of advanced prostate cancer is mediated by Mcl-1. Oncogene.

[46] Liu G, Kelly WK, Wilding G, Leopold L, Brill K, Somer B (2009). An open-label, multicenter, phase I/II study of single-agent AT-101 in men with castrate-resistant prostate cancer. Clin Cancer Res.

[47] Sonpavde G, Matveev V, Burke JM, Caton JR, Fleming MT, Hutson TE, Galsky MD, Berry WR, Karlov P, Holmlund JT, Wood BA, Brookes M, Leopold L (2012). Randomized phase II trial of docetaxel plus prednisone in combination with placebo or AT-101, an oral small molecule Bcl-2 family antagonist, as first-line therapy for metastatic castration-resistant prostate cancer. Ann Oncol.

[48] Puhr M, Santer FR, Neuwirt H, Susani M, Nemeth JA, Hobisch A, Kenner L, Culig Z (2009). Down-regulation of suppressor of cytokine signaling-3 causes prostate cancer cell death through activation of the extrinsic and intrinsic apoptosis pathways. Cancer Res.

[49] Sigl R, Wandke C, Rauch V, Kirk J, Hunt T, Geley S (2009). Loss of the mammalian APC/C activator FZR1 shortens G1 and lengthens S phase but has little effect on exit from mitosis. J Cell Sci.

[50] Deryugina EI, Quigley JP (2008). Chapter 2. Chick embryo chorioallantoic membrane models to quantify angiogenesis induced by inflammatory and tumor cells or purified effector molecules. Methods Enzymol.

