# Supplementary material for: PIAS1 is a crucial factor for prostate cancer cell survival and a valid target in docetaxel resistant cells
Source: Oncotarget. 2014 Dec 24;5(23):12043–56. doi: 10.18632/oncotarget.2658 (PMC4322998; doi:10.18632/oncotarget.2658)
Supplement: Supplementary file 1 [file oncotarget-05-12043-s001.pdf]

SUPPLEMENTARY FIGURES

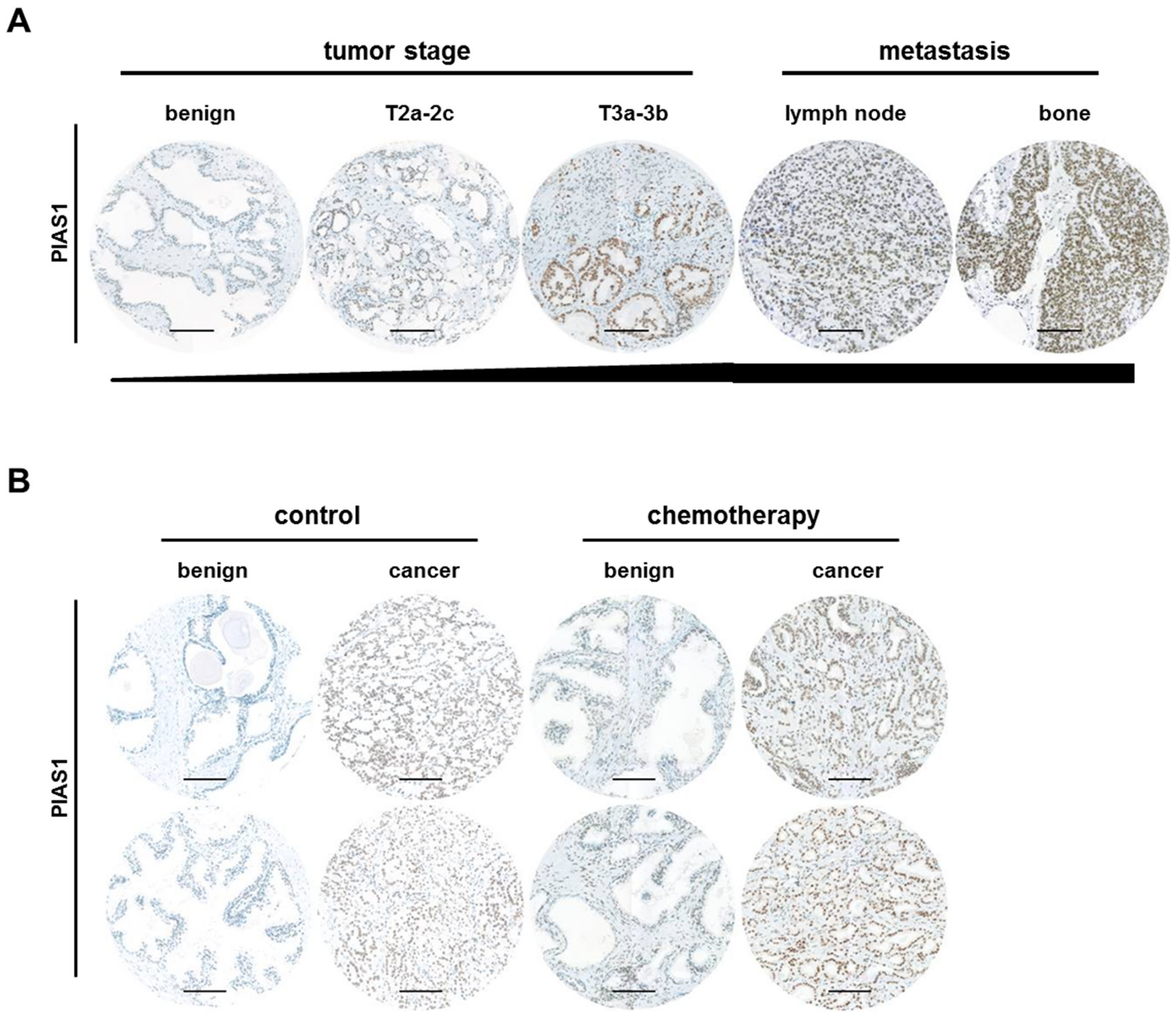

Supplementary Figure S1: Representative cores for PIAS1 expression in TMAs. Representative cores for **(A)** PIAS1 expression with increasing tumor stage and in metastatic lymph-node and bone lesions as well as for **(B)** PIAS1 expression in benign and malignant samples of control patients and of chemotherapy patients after docetaxel treatment (magnification 20x/0.5 DICII, scale bar = 100 µm).

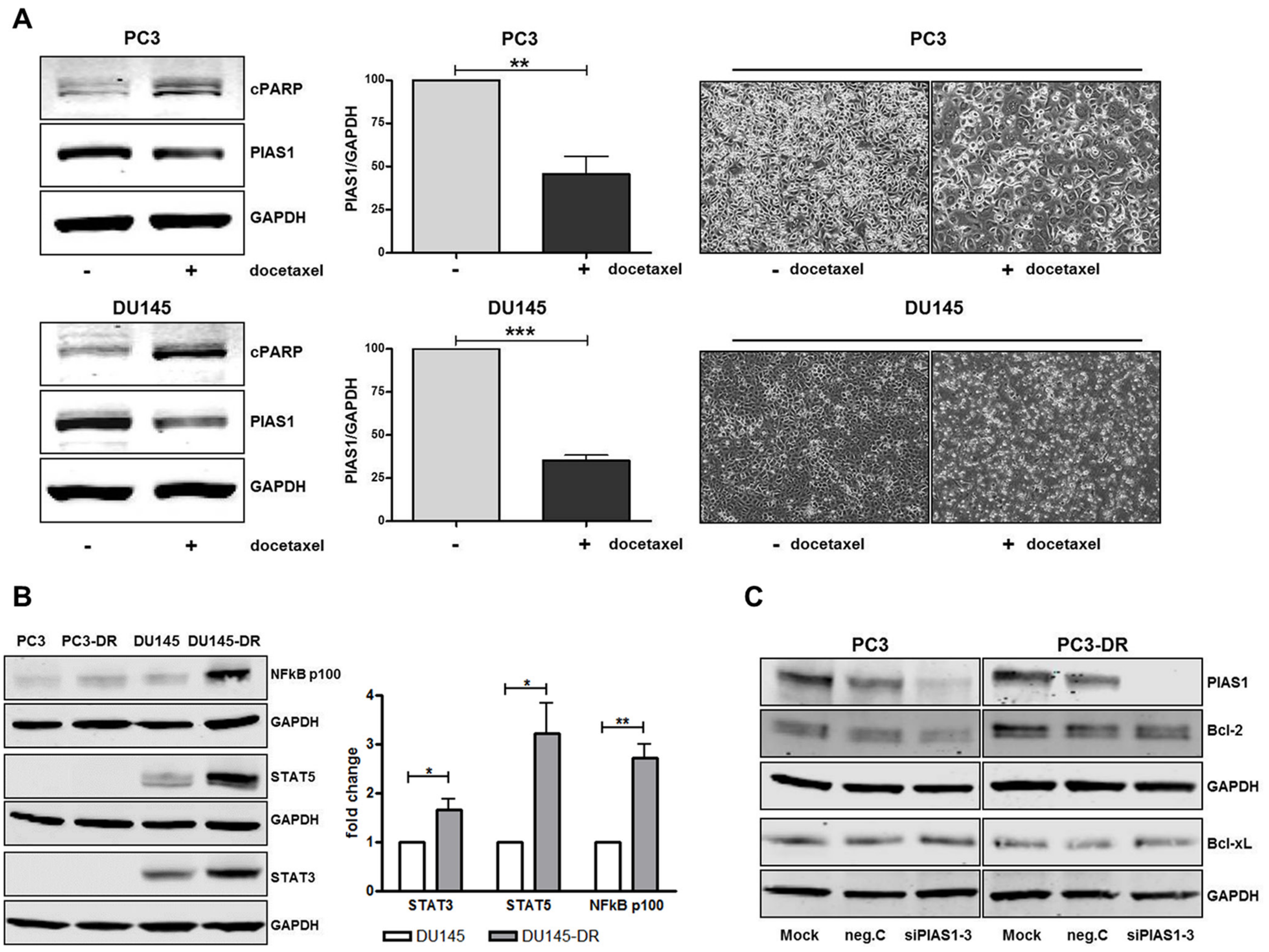

Supplementary Figure S2: Treatment specific regulation of PIAS1, STAT factors and anti-apoptotic Bcl-2 family members. **(A)** Western blot analysis and representative images of parental cells treated with 12.5 nM docetaxel for 3 days. (**, $p < 0.01$; ***, $p < 0.001$; mean + SEM). **(B)** STAT3, STAT5 and NFκB-p100 expression levels were determined in parental and docetaxel resistant cells by Western blot (*, $p < 0.05$; **, $p < 0.01$; mean + SEM). **(C)** Expression of Bcl-2 and Bcl-xL following PIAS1 downregulation for 3 days in PC3 and PC3-DR cells.

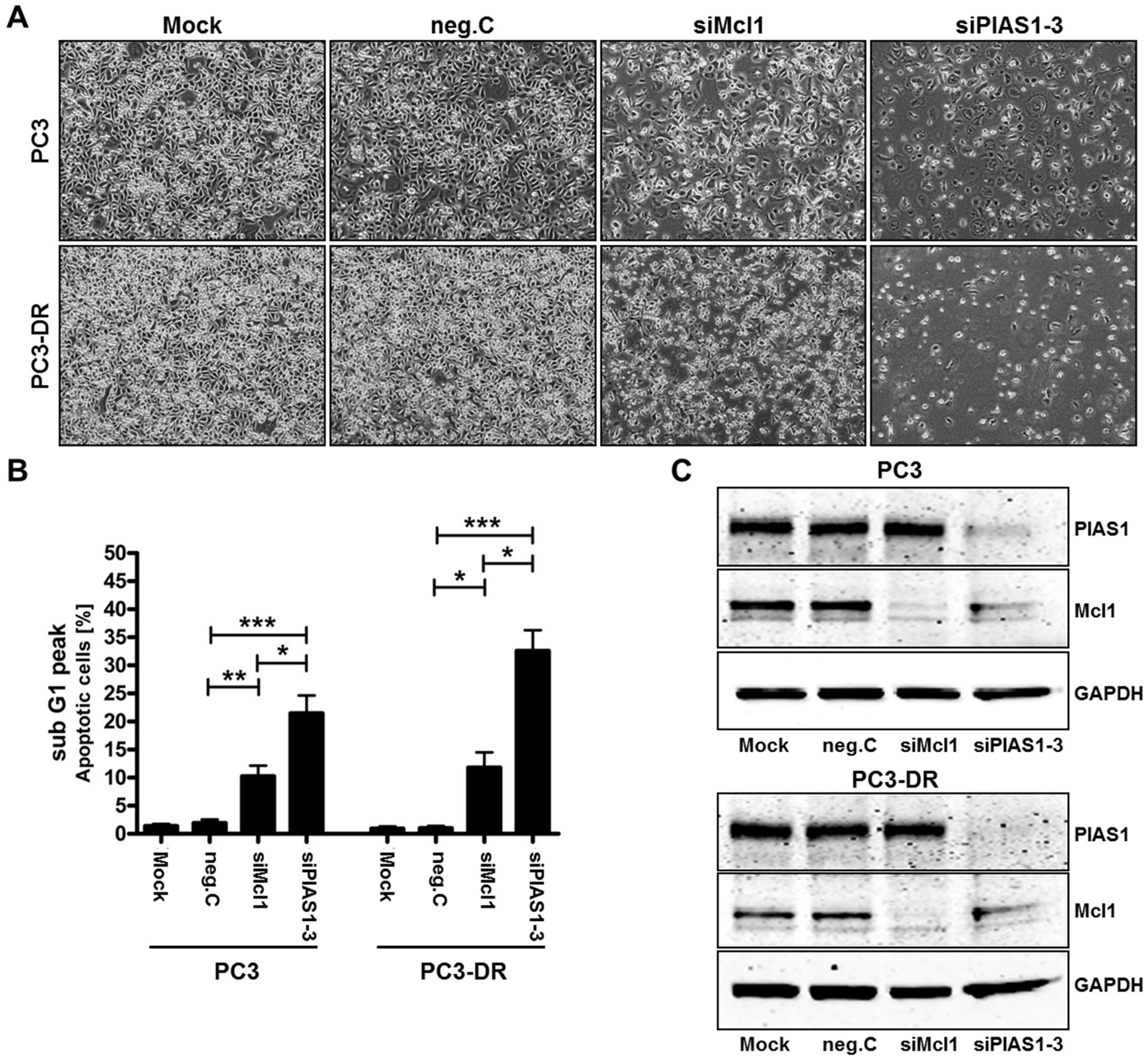

Supplementary Figure S3: Apoptotic effects of Mcl1 or PIAS1 downregulation. **(A)** Representative images and **(B)** flow cytometry analysis of PC3 and PC3-DR cells after treatment with siRNA against Mcl1 or PIAS1 for 6 days using 25 nM of siRNA (*, $p < 0.05$; **, $p < 0.01$; ***, $p < 0.001$; mean + SEM). **(C)** Representative Western blot proving PIAS1 or Mcl1 downregulation after siRNA treatment as described above.

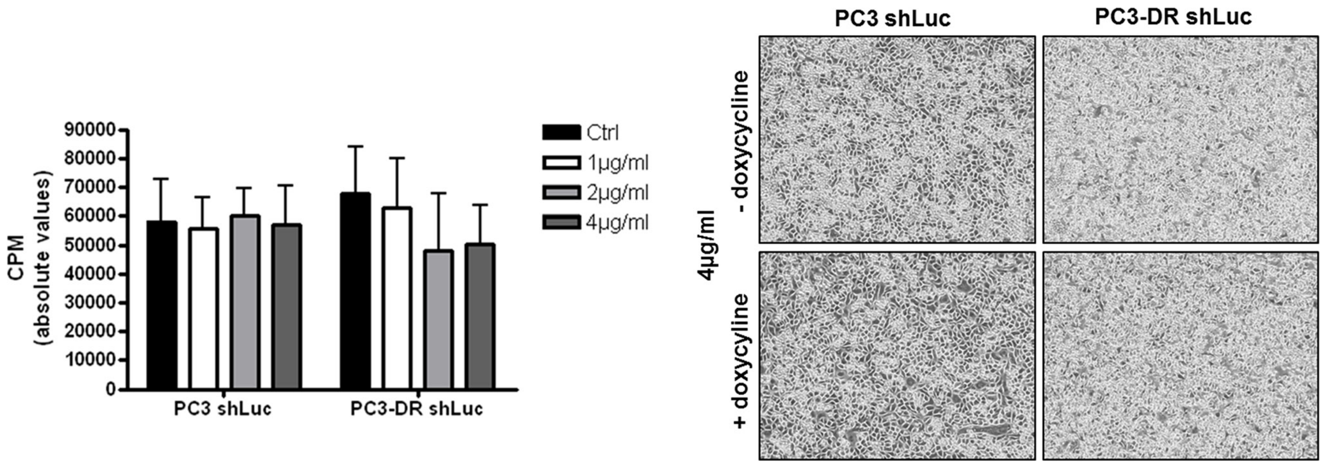

Supplementary Figure S4: Doxycycline has no influence on cell proliferation. Doxycyline has no influence on proliferation of PC3 shLuc and PC3-DR shLuc cells up to a concentration of 4 µg/ml as measured by [3H]thymidine incorporation. Representative images of PC3 shLuc and PC3-DR shluc cells in the absence or presence of 4 µg/ml doxycycline for 6 days.

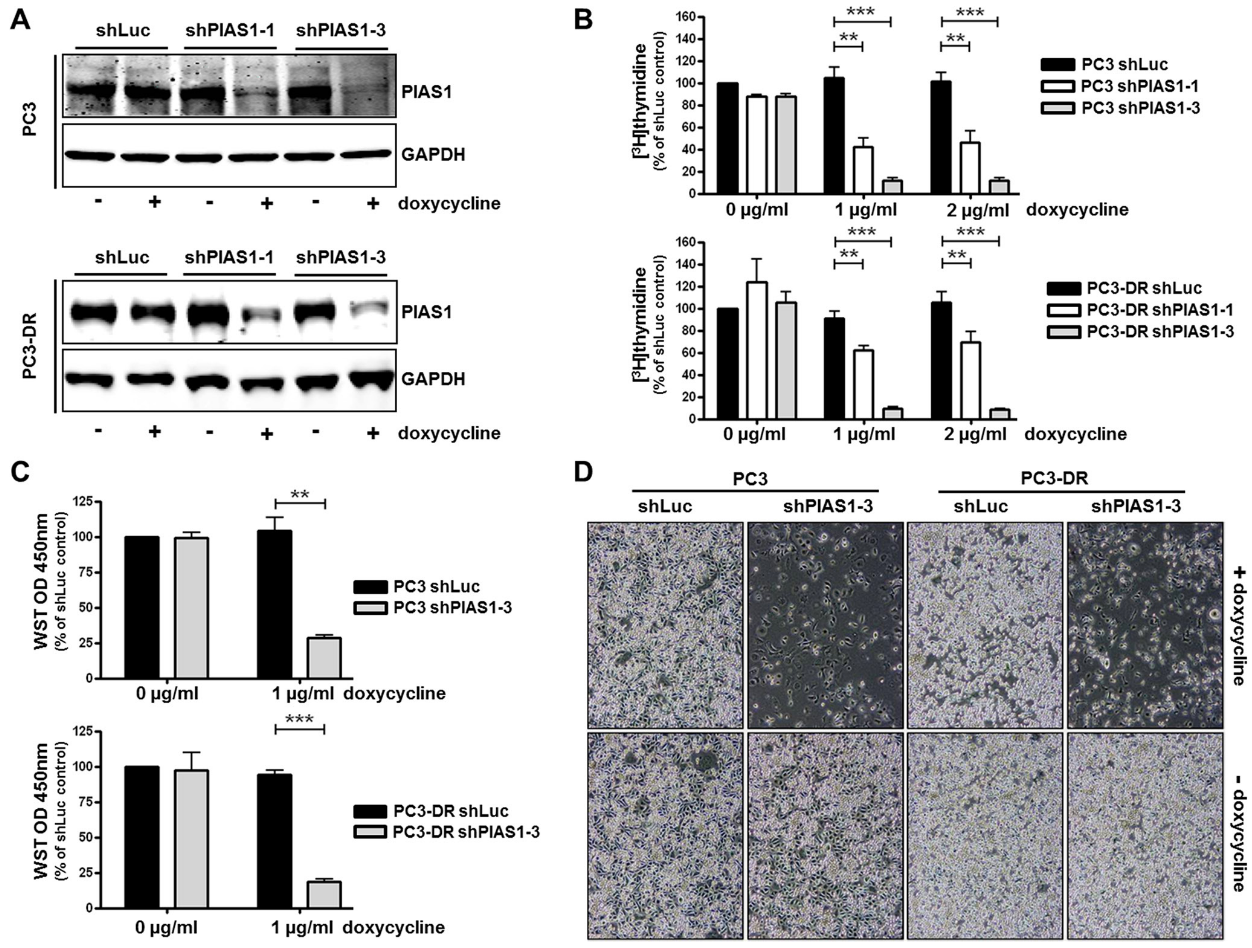

Supplementary Figure S5: Selection of 1 µg/ml doxycycline and the shPIAS1-3 sequence for *in vivo* studies. **(A)** Western blot analysis revealed that presence of 1 µg/ml doxycyline does not affect PIAS1 levels in shLuc control cells but is sufficient to reduce PIAS1 expression in shPIAS1-1 and shPIAS1-3 cells. **(B)** Proliferation was measured by [3H]thymidine incorporation after 6 days in the absence or presence of 1 µg/ml or 2 µg/ml doxycyline. Increasing concentration of doxycycline does not have additive inhibiting effects on proliferation. **(C, D)** Viability measurement by WST assay and representative pictures of PC3 and PC3-DR cells containing the shPIAS1-3 sequence in the absence or presence of 1 µg/ml doxycycline for 6 days.

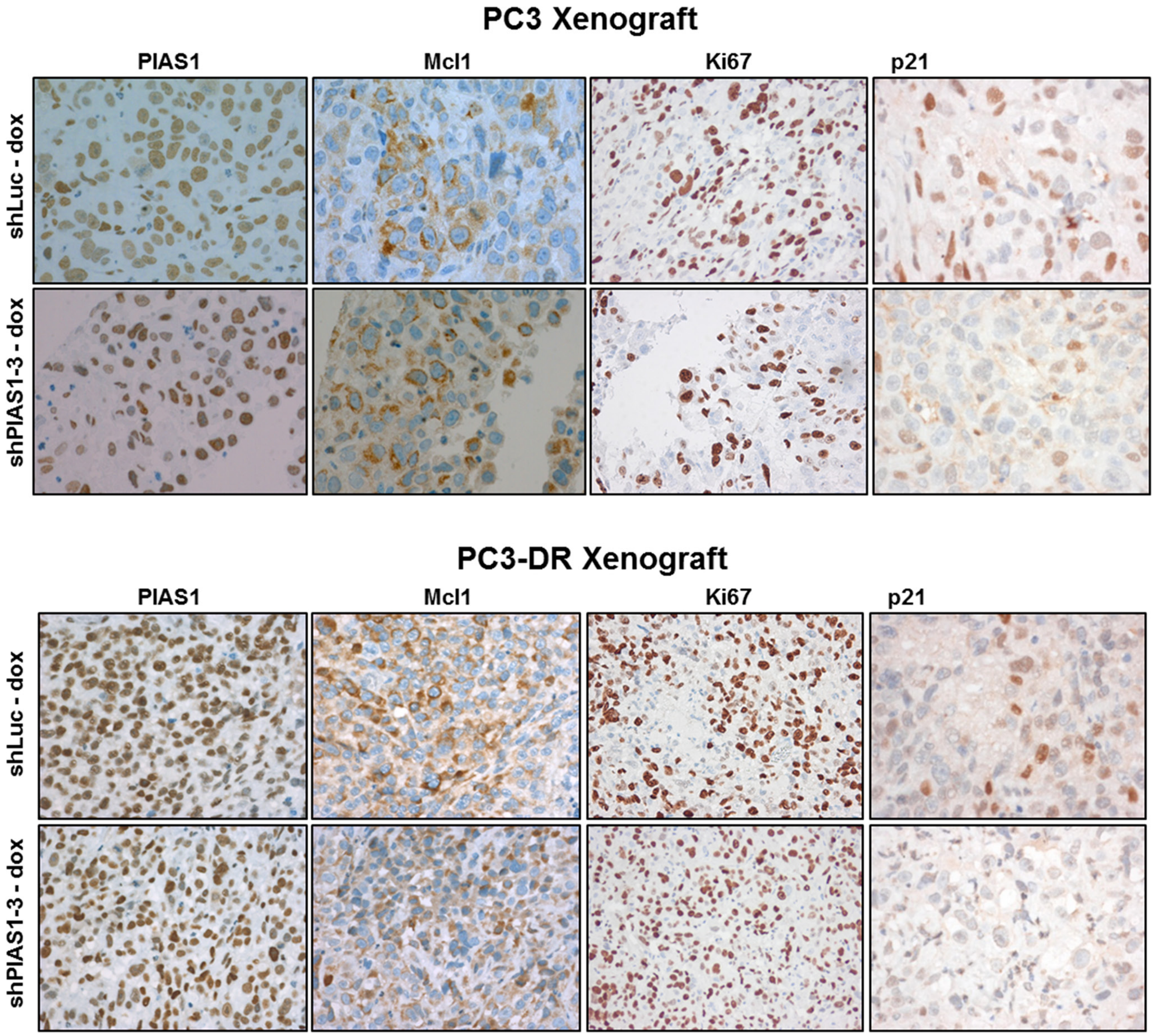

Supplementary Figure S6: PIAS1, Mcl1, Ki67 and p21 staining in the PC3 and PC3-DR xenograft control groups shLuc -dox and shPIAS1-3-dox. Representative immunohistochemistry pictures for PIAS1, Mcl1, Ki67 and p21 expression in tumors of the control groups shPIAS1-3-dox and shLuc-dox in PC3 and PC3-DR Xenografts (magnification 400x).
